# Insight into *Pelargonium odoratissimum* Essential Oil Preservative Properties Effect on Ground Beef

**DOI:** 10.3390/foods13193181

**Published:** 2024-10-07

**Authors:** Anis Ben Hsouna, Boutheina Ben Akacha, Ivana Generalić Mekinić, Natália Čmiková, Améni Ben Belgacem, Mohamed Taieb Bouteraa, Rania Ben Saad, Wissem Mnif, Maciej Ireneust Kluz, Miroslava Kačániová, Stefania Garzoli

**Affiliations:** 1 Laboratory of Biotechnology and Plant Improvement, Centre of Biotechnology of Sfax, B.P “1177”, Sfax 3018, Tunisia; benhsounanis@gmail.com (A.B.H.); akachabouthaina@gmail.com (B.B.A.); amanibelgassem@gmail.com (A.B.B.); bouteraa.taieb@gmail.com (M.T.B.); raniabensaad@gmail.com (R.B.S.); 2 Department of Environmental Sciences and Nutrition, Higher Institute of Applied Sciences and Technology of Mahdia, University of Monastir, Monastir 5000, Tunisia; 3 Department of Food Technology and Biotechnology, Faculty of Chemistry and Technology, University of Split, R. Boškovića 35, 21000 Split, Croatia; gene@ktf-split.hr; 4 Institute of Horticulture, Faculty of Horticulture, Slovak University of Agriculture, Tr. A. Hlinku 2, 949 76 Nitra, Slovakia; n.cmikova@gmail.com; 5 Faculty of Sciences of Bizerte UR13ES47, University of Carthage, BP W, Bizerte 7021, Tunisia; 6 Department of Chemistry, College of Sciences at Bisha, University of Bisha, P.O. Box 199, Bisha 61922, Saudi Arabia; wmoneef@ub.edu.sa; 7 School of Medical & Health Sciences, University of Economics and Human Sciences in Warsaw, Okopowa 59, 01 043 Warszawa, Poland; m.kluz@vizja.pl; 8 Department of Chemistry and Technologies of Drug, Sapienza University, P. le Aldo Moro, 5, 00185 Rome, Italy; stefania.garzoli@uniroma1.it

**Keywords:** ground beef preservation, antioxidant capacity, antibacterial activity, oxidative stability, food safety, shelf-life extension

## Abstract

Pelargonium plants are very popular and well-known for their essential oils (EOs), which are used for medicinal purposes and in food. This study focused on the EO of *Pelargonium odoratissimum*. First, its composition and antioxidant and antimicrobial activity were evaluated, and finally, its efficacy as a natural preservative in ground beef was tested. The main EO constituents were citronellol (40.0%), nerol (15.3%), and citronellyl formate (12.6%). The antibacterial activity of POEO showed that *Enterococcus faecalis* ATCC 29212 was the most susceptible strain compared to the other eight strains tested. The antioxidant activity, as measured by the DPPH assay, showed a dose-dependent effect with an IC_50_ comparable to the standard used, gallic acid. Aerobic plate count, psychotropic bacteria, and Enterobacteriaceae, including *Salmonella*, were reduced by the addition of *Pelargonium odoratissimum* essential oils. The oxidative stability was significantly improved compared to the untreated sample. Additionally, the results for metmyoglobin demonstrated a notable preservative effect on sensory properties, including appearance, odor, color, and overall acceptability. The ability to discriminate between all samples and correlate protein and lipid oxidation processes, microbiological characteristics, and sensory measurements was made possible by principal component analysis and heat maps. This research shows the potential benefits of using POEO in the preservation of ground beef by effectively extending shelf life and improving product safety.

## 1. Introduction

In food preservation, ensuring the longevity and safety of fresh meat is of paramount importance. Various conventional methods have been developed for this purpose, including curing, refrigeration, irradiation, chemical preservation, and bio-preservation. The addition of salt to meat effectively reduces its water activity and thereby deters spoilage microorganisms [1,2,3]. In addition, strategies such as refrigeration, supercooling, and freezing are based on lowering the temperature, which ultimately allows for easier storage and handling [4,5,6]. These preservation methods not only extend the shelf life of meat but also contribute significantly to its quality and safety. They achieve this through a variety of mechanisms: firstly, they inhibit microbial growth by creating unfavorable conditions for the proliferation of pathogens, thereby reducing the risk of spoilage and ensuring food safety; and, secondly, these methods can modulate the activity of proteolytic enzymes, which are important for the aging and tenderness of meat. By controlling enzyme activity, preservation methods help to maintain optimal texture and prevent undesirable changes. In addition, some processes can use antimicrobial agents or alter the internal environment of the meat to further increase its resistance to microbial contamination. Overall, these mechanisms help to preserve the sensory properties of the meat, including taste, color, and texture, while ensuring that the meat is safe to eat throughout its shelf life [7,8,9,10,11,12]. Despite its high efficiency in inactivating bacteria and its suitability for processing packaged meat, irradiation has negative effects on meat color due to the susceptibility of myoglobin [13,14,15]. Various preservation methods are employed to extend the shelf life and improve the quality of meat products. These include the addition of commercially available antioxidants such as butylated hydroxyanisole (BHA), butylated hydroxytoluene (BHT), and sodium nitrites and nitrates are commonly used. BHA and BHT effectively prevent oxidation by scavenging/stabilizing free radicals and inhibiting the oxidative processes that can lead to rancidity and loss of quality. These mechanisms help to maintain the freshness, flavor, and color of products over a longer period of time [1,5,16]. Essential oils (EOs) are increasingly used in food processing due to their antimicrobial and antioxidant properties, which can improve food preservation and safety. Their use is based on their ability to inhibit the growth of bacteria, fungi, and mycotoxins, thus extending the shelf life of various food products [17,18,19]. Specific applications of EOs in food preservation include their incorporation into food systems to combat spoilage caused by microbial activity. They are particularly effective in cereals, grains, fruits, and vegetables and can also play a role in active food packaging, where they help to maintain product freshness [13,20,21,22]. The natural origin of EOs makes them an alternative to synthetic components and meets consumer demand for clean-label products [23,24]. In addition, the effectiveness of EOs for food safety is enhanced by innovative techniques such as nanotechnology, which helps to encapsulate these compounds, protect them from degradation, and enable sustained release [25,26]. All these processing steps are carried out to preserve the organoleptic and aesthetic properties of the meat [27]. Consumer rejection of synthetic preservatives has led research to focus on safer and more natural products as preservatives. This search in meat processing is currently focused on products of plant origin, especially EOs [27].

This study represents a novel investigation into the use of the EO of *Pelargonium odoratissimum* (L.) (*PO*) for the preservation of ground beef. Based on a survey of the literature, this is the first study investigating the effects of *PO* on the quality and safety of ground beef, representing an innovative approach to food preservation as only a few studies have to date investigated the potential of *Pelargonium graveolens* application in foods [28]. The current study fills this gap by providing new insights into the preservative effects of *PO* and highlighting its potential as a natural alternative to synthetic additives [29]. The present study aimed to determine the chemistry of POEO as well as its antioxidant and antibacterial effects in different in vitro systems. In addition, the preservative effect of POEO was evaluated in situ by studying its effects on the physicochemical, microbiological, and sensory properties of minced meat. By examining these parameters, the study shows how POEO can improve the quality of meat and extend its shelf life. The antioxidant properties of POEO help protect against lipid oxidation, while its antibacterial properties ensure the safety of the meat. These findings are significant given the lack of studies on the use of this EO in food.

## 2. Materials and Methods

### 2.1. Sample

The *Pelargonium odoratissimum* EO (POEO) isolated by steam distillation from the flowers and leaves of the plants growing in Tuscany (Italy, harvested in July 2022), was provided by “Essenziale” Azienda Agricola, San Donato in Poggio (FI), Italy.

### 2.2. GC-MS Analyses of EO

A gas chromatograph (model Clarus 500, Perkin Elmer, Waltham, MA, USA) coupled to a mass spectrometer and equipped with a flame ionization detector was used for POEO analysis, while separation was performed on a Stabilwax fused-silica capillary column (Restek, Bellefonte, PA, USA) (60 m × 0.25 mm, 0.5 mm) The temperature program of the oven started from 60 °C up to 220 °C for 20 min at a rate of 6 °C/min. Helium was used as the carrier gas with a flow rate of 1.0 mL/min. Mass spectra (MS) were recorded by electron impact ionization at 70 eV in full scan mode (mass range of 35–550 m/z). Compounds were identified by comparing the obtained MS with the Wiley 2.2 (Wiley, New York, NY, USA) and Nist 11 (Gaithersburg, MD, USA) databases and by calculating the linear retention indices (LRIs) using a C_8_–C_25_ *n*-alkanes mixture, which were then compared with reference values from the literature. Relative compound content was determined as an average percentage based on total peak area, normalizing without the use of an internal standard or correction factors. All analyses were performed in triplicate [14].

### 2.3. Antioxidant and Antibacterial Activity

#### 2.3.1. DPPH Assay

The DPPH test was performed as previously described [14,30]. In brief, 2 mL of various dilutions of POEO were mixed with 2 mL of 2,2-diphenyl-1-picrylhydrazyl (DPPH) solution (0.1 mM). After 30 min, the absorbance of the samples was measured spectrophotometrically at 517 nm (Jasco 7800, Jasco Corporation, Tokyo, Japan). Gallic acid was used as a reference standard [31].

The IC_50_, the POEO concentration leading to 50% inhibition, was determined by plotting the percentage inhibition of radicals against the POEO concentration. To ensure reliability and consistency, all experiments were performed in triplicate

#### 2.3.2. Determination of Inhibition Diameters

The authentic pure bacterial cultures were sourced from international culture collections, specifically the American Type Culture Collection (ATCC), and from the local culture collection of the Centre of Biotechnology of Sfax, Tunisia. The bacterial strains consisted of Gram-positive species *Bacillus cereus* (*B. cereus*) (ATCC 14579), *Staphylococcus aureus* (*S. aureus*) (ATCC 25923), *Enterococcus faecalis* (*E. faecalis*) (ATCC 29212), *Micrococcus luteus* (*M. luteus*) (ATCC 1880), and *Listeria monocytogenes* (*L. monocytogenes*) (ATCC 19117), as well as Gram-negative species *Salmonella enterica* (*S. enterica*) (ATCC 43972), *Escherichia coli* (*E. coli*) (ATCC 25922), and *Pseudomonas aeruginosa* (*P. aeruginosa*) (ATCC 9027). The bacteria were cultured on Mueller–Hinton agar (MH) at 37 °C for 12–24 h, with the exception of the *Bacillus* species, which were incubated at 30 °C [5].

The antimicrobial activity of POEO was assessed using the agar-well diffusion technique, following the method outlined by Sebei et al. [32]. In this procedure, 15 mL of molten agar (at 45 °C) was poured into sterile 90 mm Petri dishes. Once the agar had set, 100 μL of the prepared microbial cell suspension was spread evenly over the surface of Mueller–Hinton (MH) agar plates (Oxoid Ltd., Basingstoke, UK). Subsequently, wells with a diameter of 6 mm were carefully created using a sterile Pasteur pipette.

POEO was dissolved in dimethyl sulfoxide (DMSO, 10%, *v*/*v*) to a final concentration of 25 and 50 mg/mL and filtered using black polycarbonate filters (0.22 μm, Millipore Burlington, MA, USA). Thus, 50 μL of the sample was added to the wells, and the plates were incubated at 37 °C for 24 h. Carbenicillin (10 and 5 μg/well) was used as a positive control and DMSO as a negative control. Tests were performed in triplicate and antimicrobial activity was expressed as the diameter (in mm) of the circular zones of inhibition around the well.

#### 2.3.3. Determination of Minimum Inhibitory Concentrations

The minimum inhibitory concentrations (MIC) of POEO were determined against the eight pathogenic strains of bacteria [33]. The final concentrations were 30%, 15%, 7.5%, 3.75%, 1.87%, 0.93%, 0.46%, 0.23%, 0.11%, 0.058%, 0.029%, and 0.014% *v*/*v*. Subsequently, 10 μL of the cell suspension containing 10^6^ colony-forming units (CFU)/mL was added to each well. After incubation, the wells were treated with 0.5 thiazolyl blue tetrazolium bromide (MTT, 0.5 mg/mL) and incubated at 37 °C for 30 min. The mixture in the wells remained clear after the addition of MTT, demonstrating cessation of microbial growth.

MH broth and water were negative controls while the bacterial inoculum and MH were used as positive controls. The MIC was reported as the sample concentration in the well where no visible bacterial growth was observed [33].

#### 2.3.4. Determination of Minimum Bactericidal Concentrations

The minimum bactericidal concentrations (MBCs) were detected from the last three wells containing the sample in which no visible bacterial growth was detected after incubation of the plate [33]. For this purpose, 10 µL of the well contents were inoculated onto MH plates which were then incubated overnight at 37 °C, and the development of colonies at each inoculation site was observed. The position where no bacterial colonies developed is referred to as MBC [34].

### 2.4. Analysis Conditions for Minced Ground Beef Samples

Minced raw beef (*Bos taurus*) from the chuck cut, initially ground with a 10 mm disc and then with an 8 mm disc, was obtained from a local market in Sfax, Tunisia. The meat was transported to the laboratory within one hour and stored on ice in an insulated polystyrene container. For storage, the minced beef was divided into four equal portions, each of which was placed in a sterile plastic bag. The meat samples were divided into five batches: one negative control (untreated minced meat), one positive control (0.01% BHT), and three batches treated with essential oils (EO): 1POEO (0.25%), 2POEO (0.5%), and 4POEO (1%). The essential oils were added to the minced meat under a microbiological hood, with the amount of EO adjusted based on meat weight (*w*/*w*) to ensure accurate dosing. The preservatives were added during the grinding process to ensure even distribution. After preparation, the samples were stored at 4 °C for 0, 3, 7, 10, and 14 days in standard plastic bags without protective gas packaging. For each experiment, all tests were performed on a batch of raw ground beef [35].

### 2.5. Microbial Load Changes

To assess the levels of aerobic bacteria (APC), psychrotrophic bacteria (PTC), and Enterobacteriaceae, 25 g of each sample was homogenized in 225 mL of a sterile 0.85% NaCl solution for 10 min [36,37]. Serial decimal dilutions of the homogenized samples were prepared and plated on the appropriate media for microbiological analysis. APC was quantified on plate count agar (PCA, Oxoid Ltd., Basingstoke, UK) and incubated at 30 °C for 48 h. PTC was determined similarly to APC, but the plates were incubated at 7 °C for 10 days. The number of Enterobacteriaceae was calculated using Violet Red Bile Glucose Agar (VRBG, Oxoid Ltd.) and incubated at 37 °C for 24 h. *Salmonella* spp. was determined using xylose-lysine deoxycholate (XLD, Oxoid Ltd.) for 24 h. Microbiological counts were expressed in log CFU/g [35,38].

### 2.6. Physicochemical Changes

#### 2.6.1. pH Analysis

Five grams of the meat samples were mixed with 50 mL of distilled water. The resulting suspension was homogenized for 30 s (13,000 rpm) and then filtered. The pH of the filtrate was recorded using a pH meter (model YK-21PH, Lutron, Taiwan) [16].

#### 2.6.2. Protein and Lipid Oxidation Analysis

Metmyoglobin (MetMb) was extracted and quantified according to the method reported by Krzywicki [39]. Five grams of each sample were homogenized with 25 mL of 0.04 M K_3_PO_4_ buffer (pH 6.8). The homogenates were incubated in an ice bath (CB-200, Cole-Parmer, Cambridgeshire, UK) for 1 h to ensure complete extraction, and then centrifuged at 4500× *g* for 30 min (CDL7M, Changsha Yingtai Instrument Co., Ltd., Changsha City, China). The absorbance of the supernatant was recorded at 525, 572, and 700 nm. The MetMb percentage was calculated according to the following formula by Elhadef et al. [40]:

% MetMb = (−2.51(A_525_/A_572_) + 0.777(A_525_/A_700_) + 0.8(A_572_/A_700_) + 1.098) × 100 (1)

where A_525_, A_572_, and A_700_ are the absorbances recorded at 525 nm, 572 nm, and 700 nm, respectively.

The procedure for determining the reactive substances of thiobarbituric acid (TBARS) has already been described by Eymard et al. [41]. In this method, 100 µL of BHT (1 g/L in ethanol) and 16 mL of trichloroacetic acid (TCA, 50 g/L) were added to 2 g of the sample. The mixture was homogenized for 15 s at 20,000 rpm and then filtered. Two milliliters of the filtrate (or 2 mL of TCA for the blank) was then mixed with 2 mL of thiobarbituric acid solution (20 mmol/L). The sealed tubes were heated to 70 °C for 30 min and then rapidly cooled on ice. The absorbance was measured at 508 nm (A_508_), 532 nm (A_532_), and 600 nm (A_600_) compared to the blank, with the absorbance at 532 nm corrected for baseline deviation as follows:

A_532_ corrected = A_532_ − [(A_508_ − A_600_) × (A_600_ − A_532_)/(A_600_/A_508_)] − A_600_ (2)

The results were expressed in mg of malonaldehyde (MDA) per kg of meat.

### 2.7. Sensory Evaluation

Thirty knowledgeable individuals from our department, consisting of both faculty members and graduate students, participated in the sensory evaluation of the meat. The pre-training evaluations were in accordance with the American Meat Science Association guidelines [9]. A panel of thirty evaluators was trained to rate characteristics such as color, appearance, odor, and overall acceptability of the beef using a five-point scale for scoring: 1 = very unacceptable; 2 = unacceptable; 3 = neither acceptable nor unacceptable; 4 = acceptable; and 5 = very acceptable. The results are presented based on the panelist’s predominant ratings.

### 2.8. Statistical Analysis

All results were expressed as mean ± standard deviation (SD) (n ≥ 3). Samples were analyzed after 0, 3, 7, 10, and 14 days of storage, with three replicates for each day. A two-way analysis of variance (ANOVA) was performed for all variables. Statistical significance of mean differences was assessed using triplicate measurements, and a Tukey test was performed using GraphPad Prism 9.0 for Windows (InStata, GraphPad Software, Boston, MA, USA). Correlations were established to analyze the relationships between different types of parameters, namely microbiological, biochemical, and sensory characteristics, in order to shed light on the meat degradation process during storage and to understand how these parameters interact with each other.

Principal component analysis (PCA) was conducted using GraphPad Prism 9.0 (GraphPad Software, Boston, MA, USA) to visualize the progression of treatment scores over time and to classify meat samples according to microbial counts, physicochemical properties, and sensory attributes. Heat maps were created for each group using a quadratic Euclidean distance matrix and the Ward clustering method, which generated dendrograms for all samples.

## 3. Results

### 3.1. GC-MS Analyses

The GC-MS analyses conducted allowed the detection of twenty-five compounds in POEO, which was rich in oxygenated monoterpenes (81.2%), while sesquiterpenes accounted for only 11%. The dominant compounds were citronellol (40.0%), nerol (15.3%), and citronellyl formate (12.6%) followed by (-)-aristolene (9.8%), isomenthone (7.4%), linalool (4.8%), and geranyl isobutyrate (2.6%). In addition, several minor compounds with proportions between 0.1 and 1.3% were detected (Table 1).

The results are consistent with previous reports in which citronellol was the major compound, followed by other components such as citronellyl formate, geraniol, isomenthone, and linalool [29].

Citronellal is used as a raw material, as an insect repellent, in herbal medicine, aromatherapy, perfumery, cosmetology, and food. It is recognized for its sedative action on the nervous system and its antinociceptive, antioxidant, biopesticide/repellent/antimicrobial, and fungicidal effects. In mice, citronellal injection inhibits pain sensitivity (analgesia) and reduces spontaneous activity (sedative). Sleep time is significantly increased [42]. α-Phenylseleno citronellal and the natural terpenoid R-citronellal have an antidepressant effect in mice [43]. It is considered a repellent for biting insects (mosquitoes) but also has some effectiveness against ticks [44]. Perfumes based on citral, citronellal, or geraniol are perfectly suitable for deodorizing feet due to their antimicrobial effect [45]. Citronellal damages the membranes of microscopic fungi such as *Penicillium* and *Candida* [43]. It is a proven fungicide against two rice pathogens, *Rhizoctonia solani* and *Helminthosporium oryzae* [46]. In combination with cinnamaldehyde, it is active against *Penicillium digitatum*, which is responsible for the green mold of citrus fruits for which it is a natural preservative with a minimum dosage of 4 mL per liter [46]. Its antioxidant potential is low, lower than that of ascorbic acid [45]. Citronellal improves endothelial dysfunction and reduces atherosclerotic plaques in rats by reducing oxidative stress [47].

In general, there are many factors that influence the yield and phytochemical profile of EO, which are often related to the environmental and growth conditions of the plant, the plant part used, and the extraction method applied [34]. Climatic and geographical conditions have been shown to influence the performance of key essential oil components. Thus, EOs rich in citronellol, nerol, geraniol, and menthone were mostly extracted from plants grown at high altitudes and in a temperate climate, while oils rich in isomenthone, linalool, and citronellyl formate were extracted from plants grown at lower altitudes [35].

### 3.2. Antibacterial Activity

**Figure 1:**
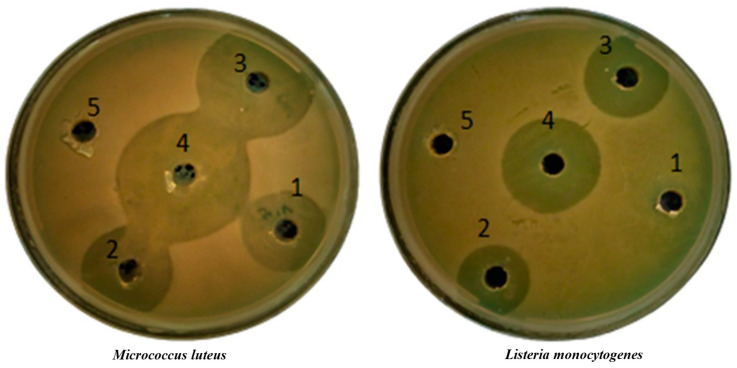
Image of inhibition zones of *M. luteus* and *L. monocytogenes* with carbenicillin and POEO treatment. (1): POEO = 25 mg/mL; (2) Carbenicillin = 10 µg/mL; (3) Carbenicillin = 5 µg/mL; (4) POEO = 50 mg/mL; (5) DMSO as negative control.

EOs are mixtures of aromatic volatile compounds that play a key role in protecting plants against bacteria, viruses, fungi, and pests [22]. In this study, the antibacterial efficacy of POEO against major food-contaminating bacteria, namely, *B. cereus*, *S. aureus*, *E. faecalis*, *M. luteus*, *L. monocytogenes*, *P. aeruginosa*, *E. coli*, and *S. enterica* was evaluated by in vitro experiments employing the inhibition diameter method, and determination of MIC and MBC. The results are shown in Figure 1 and Table 2 and Table 3.

Based on the reported values for the zone diameters of inhibition, the results were classified as follows: not sensitive (-) for zone diameters of 8 mm or less; sensitive (+) between 8 and 14 mm; very sensitive (++) between 14 and 20 mm; and extremely sensitive (+++) for 20 mm or more [48]. As can be seen from the results, the antibacterial activity of POEO was tested at two different concentrations, 15 and 50 mg/L, respectively. The EO was not effective against *B. cereus*, *P. aeruginosa*, and *S. enterica* at either concentration tested, while it only achieved an inhibition diameter of 21 mm against *L. monocytogenes* at a concentration of 50 mg/mL. The only Gram-negative bacterium that was sensitive to POEO was *E. coli*, while among the Gram-positive bacteria, *M. luteus* showed the greatest sensitivity, with an inhibition zone of 29 mm at a concentration of 50 mg/mL. Interestingly, the effectiveness of POEO did not vary significantly between the two concentrations tested against *S. aureus*, although significantly higher inhibition for all other bacterial species was observed at the higher POEO concentration. For the sake of clarity, the bacteria tested were categorized as Gram-positive (*M. luteus*, *S. aureus*, and *E. faecalis*) and Gram-negative (*E. coli*). The different bacterial strains showed different sensitivities to carbenicillin, the reference antibiotic, with Gram-negative bacteria generally showing smaller zones of inhibition than Gram-positive bacteria, indicating lower susceptibility. Carbenicillin at 10 µg/mL was most effective against *E. coli* with a zone of inhibition of 31 mm, while *E. faecalis* was most sensitive among the Gram-positive bacteria with a zone of inhibition of 29 mm. Antibacterial activity was measured at 37 °C, a temperature that mimics physiological conditions.

The reported results are consistent with those of Andrade et al. [29], who showed that POEO was able to completely inhibit fungal growth, but had little effect on *S. aureus* ATCC 25923 and *E. coli* ATCC 25992.

To verify the inhibitory efficacy of POEO against these pathogenic strains, we performed a dilution test in a liquid medium to determine the minimum inhibitory concentration (MIC) and minimum bactericidal concentration (MBC). POEO was found to be effective against all bacterial strains tested with MIC values between 5.6 and 9.35 mg/mL, with the exception of *P. aeruginosa*, where the MIC value was >30 mg/mL (Table 3). The lowest MIC was detected for *E. faecalis*, where a bacteriostatic effect was achieved, followed by *M. luteus*, which had the lowest MBC value (8.25 mg/mL). Among Gram-negative species, *E. coli* was, as expected, more sensitive than *S. enterica* with MIC values of 8.25 and 9.35 mg/mL, respectively.

The reference antibiotic, carbenicillin, was found to be effective against strains with MIC values below those of POEO, which were between 0.92 and 3.12 mg/mL. Of note, carbenicillin consistently had lower MIC and MBC values compared to POEO, highlighting its superior antibacterial potency against the strains tested. While both POEO and carbenicillin exhibited bactericidal activity against most strains, nuanced differences were observed, particularly when interpreting activity against specific strains such as *E. faecalis* and *Listeria monocytogenes.* According to the MBC/MIC ratio, POEO showed its efficacy as a bactericidal agent, killing the bacteria present in the inoculum. In general, EOs are characterized by many components and their mode of action probably involves several targets in the bacterial cell. The hydrophobic property of EOs allows them to disperse in the lipids of cell membranes and mitochondria, making them permeable and causing the leakage of cellular components. Parameters that increase the activity of EOs are low pH, temperature, and oxygen concentration [46,49,50].

POEO has strong antibacterial properties, largely due to its complex chemical composition, which contains key compounds such as methyl eugenol, citronellol, geraniol, and linalool. These components work synergistically together to enhance the antimicrobial efficacy of the oil, making it effective against a broad spectrum of pathogens. This is consistent with in vitro susceptibility testing, where POEO has shown significant antibacterial activity, particularly against Gram-positive *S. aureus*, with an MIC of 500 µg/mL [32]. In addition to its essential oil, *PO* is also promising for antimicrobial applications in other forms, such as extracts. For example, in one study, zinc oxide nanoparticles were synthesized using an aqueous extract of *PO* leaves as a reducing agent. These nanoparticles were tested against *S. aureus*, *P. aeruginosa*, and *E. coli* using the disc diffusion method, which confirmed their antibacterial efficacy [51].

The therapeutic potential of *PO* is, therefore, underlined by its ability to act in a comparable way to conventional antibiotics in combating bacterial strains. Whether used as an essential oil or in the form of an extract, the bioactive components and innovative applications such as the synthesis of nanoparticles underline its importance for antimicrobial research.

### 3.3. Antioxidant Activity

The DPPH assay determines the ability of the samples to scavenge free radicals. The results show that gallic acid with an IC_50_ value of 31.44 µg/mL has a more than five times higher radical scavenging capacity than POEO with an IC_50_ value of 164.24 µg/mL (*p* < 0.05). The reducing effect of POEO is likely due to citronellol, an acyclic monoterpenoid identified as the main component of POEO. Citronellol contributes to the antioxidant effect via several mechanisms. Primarily, citronellol acts as a hydrogen donor and neutralizes free radicals, as used in the DPPH assay. By donating hydrogen atoms, citronellol stabilizes and deactivates the free radicals and prevents them from causing oxidative damage [52]. This hydrogen donation interrupts the chain reaction of oxidative processes and reduces overall oxidative stress [53]. In addition, citronellol can also chelate metal ions, which are catalysts for radical formation, further enhancing its antioxidant properties. This mechanism of action is consistent with the high antioxidant activity reported for citronellol-containing essential oils, such as *Pelargonium graveolens* EO, which has a remarkable IC_50_ value of 0.802 mg/mL, reflecting its significant free radical scavenging ability, which is also characterized by the presence of citronellol (24.54%) as a major constituent [52]. Our results are in agreement with those of Prakash et al. [18], who reported an IC_50_ of POEO of 96.63 μL/mL. The low IC_50_ values of the tested EOs support their use as valid plant antioxidants that can improve the shelf life of foods by combating free radicals and oxidation of unsaturated lipids.

### 3.4. Effect of POEO on Conservation of Raw Minced Beef Meat

#### Changes in Microbial Status of Meat Samples

**Figure 2:**
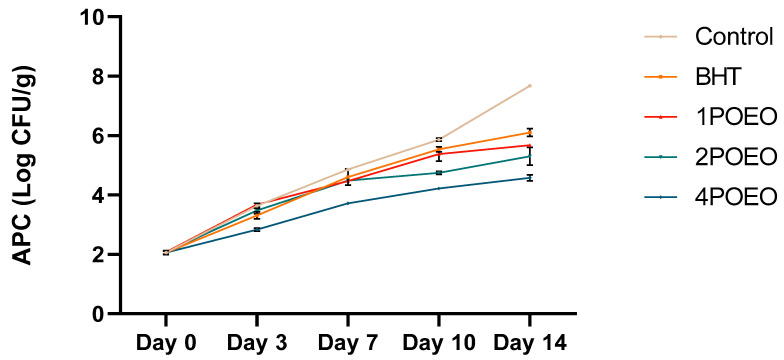
Effect of POEO on microbiological qualities of raw minced ground beef APC. Values are the mean of three individual replicates (means ± SD).

In meat production, maintaining food safety and extending shelf life are of paramount importance and are directly related to microbial activity [54,55,56,57,58,59]. As expected, there was a significant increase in aerobic plate counts (APC) during prolonged storage, indicating microbial proliferation. This trend was consistent under the different processing conditions and highlights the importance of storage time for the dynamics of microbial growth. Figure 2 illustrates the critical role of storage time in determining food safety and shelf life.

POEO effectively reduced the total number of bacteria in ground meat (Figure 2). The values of all microbial indicators were strongly affected by the addition of the synthetic antioxidant BHT and three different POEO concentrations, with APC values around day 14 of 7.65, 5.96, 5.66, 4.97, and 3.98 log_10_ CFU/g for the control, BHT, 1POEO, 2POEO, and 4POEO, respectively. These results show that the preservative effect of POEO is dose-dependent, with similar effects for BHT and POEO, and the best effect for POEO was obtained with a minimum shelf-life extension of 8 days.

**Figure 3:**
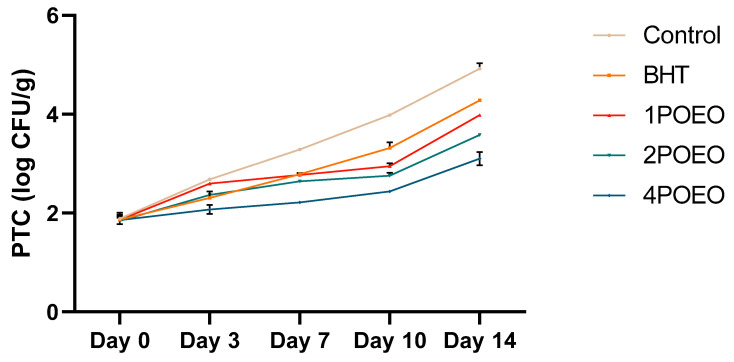
Effect of POEO on microbiological qualities of raw minced ground beef PTC. Values are the mean of three individual replicates (means ± SD).

A significant increase in PTC over time was observed in ground beef (Figure 3). However, the PTC increase in POEO-treated samples was significantly lower than in untreated and BHT-treated samples, with values of 3.00 log_10_ CFU/g for 4POEO compared to 4.84 and 4.26 log_10_ CFU/g for the control and BHT, respectively. PTC is the best-known microbial flora of meat and meat products preserved under cold conditions.

These results are in agreement with those of Ben Akacha et al. [27], who tested the effect of the EO of *Lobularia maritima* in ground beef. This treatment significantly reduced the contaminating flora of the meat (APC and PTC). The maximum PTC value was only reached on the 14th day of storage at 4 °C.

EOs have been shown to possess antibacterial and antifungal effects against various meat-associated microorganisms, including bacteria [34,35]. Many recent studies have investigated the effects of plant EOs, alone or in combination with other EOs and/or preservation methods, on meat and meat products [17,35,36], due to the better stability, microbiological safety, and aroma effects of EOs.

Enterobacteriaceae are generally used as hygiene indicators to monitor the effectiveness of preventive measures [56]. The results of Enterobacteriaceae and *Salmonella* spp. counts showed that the initial bacterial count was <1 log_10_ CFU/g (Table 4). The results showed a significant difference (*p* < 0.05) in Enterobacteriaceae and *Salmonella* spp. over storage time in the same samples, except for POEO-containing samples at 10–14 days of cold storage. The results of this study demonstrate the efficacy of POEO treatment in limiting the proliferation of Enterobacteriaceae compared to control and BHT. This underlines the potential of POEO as a natural antimicrobial agent for the preservation of meat products during cold storage and offers a promising alternative to synthetic additives such as BHT. These results are consistent with those obtained by adding *Mentha spicata* EO at three different concentrations (0.5, 1.0, and 1.5% *v*/*w*) to raw camel meat to extend shelf life and investigate the inhibitory effect against *L. monocytogenes* [38,39]. Based on these results, POEO offers a natural and sustainable alternative to synthetic preservatives, thus meeting consumer preferences for clean-label and minimally processed foods. The bio-preservative effect of POEO enables the preservation of food products without artificial additives, improving their safety and quality while meeting the demand for natural and healthy food.

### 3.5. Physiochemical Analysis

#### Changes in MetMb and TBARS of Raw Ground Beef Samples

**Figure 4:**
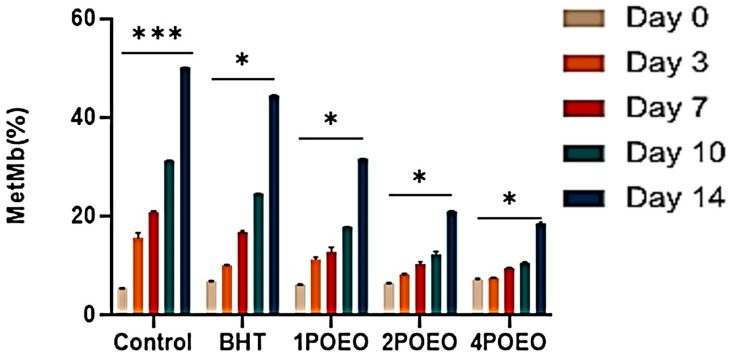
Changes in biochemical parameters of raw ground beef treated with different POEO concentrations during refrigerated storage for 14 days: changes in MetMb (%). Values are the mean of three individual replicates (means ± SD). *** *p* ≤ 0.001, * *p* ≤ 0.05 for the same concentration are significantly different.

**Figure 5:**
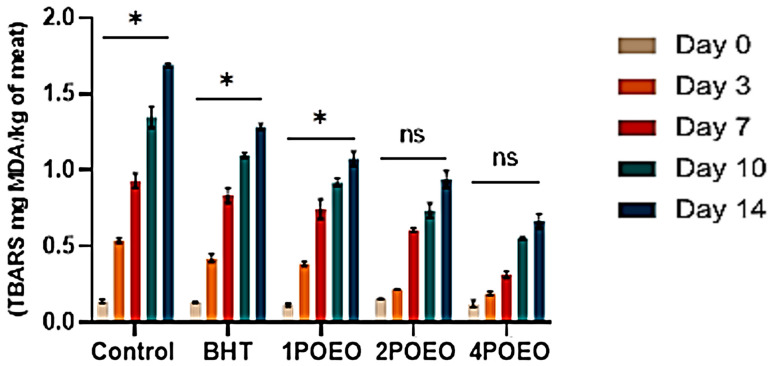
Changes in biochemical parameters of raw ground beef treated with different POEO concentrations during refrigerated storage for 14 days: changes in TBARS (mg MDA/kg of meat). Values are the mean of three individual replicates (means ± SD). (ns) non-significant, * *p* ≤ 0.05 for the same concentration are significantly different.

Heme pigments, including nitric oxide ferric hemoglobin, carbon monoxide ferric hemoglobin, methemoglobin, and especially metmyoglobin (MetMb), play a key role in catalyzing lipid oxidation in both raw and cooked meat. These pigments are usually present in their reduced form, e.g., as oxymyoglobin (OxyMb) and deoxymyoglobin, although it is still unclear whether heme iron directly affects lipid oxidation [60]. Nonetheless, hemoglobin and myoglobin forms can be induced, and iron-free heme proteins can initiate self-oxidation [15,61,62]. Figure 4 and Figure 5 illustrate the effects of POEO on the biochemical properties of raw ground beef.

Figure 4 shows the development of MetMb content over 14 days of storage at 4 °C. All treatment groups showed lower MetMb content compared to the control group, with 4POEO (14.8%) having the lowest level (*p* < 0.05). The results show that the synergy of POEO constituents effectively delays the formation of MetMb. Meat discoloration during cold storage is usually associated with the accumulation of MetMb on the sample surface.

During cold storage, the accumulation and discoloration of MetMb strongly depend on the presence of lipids and the oxidation–reduction system [61]. Primary products of lipid oxidation such as hydroperoxides and reactive oxygen species are known to oxidize ferrous ions in OxyMb to ferric ions present in MetMb [62].

The results of this study show that POEO effectively inhibits the oxidation of myoglobin in ground beef and suggest for the first time its application in this context. TBARS are an indicator of secondary oxidation products, particularly hydrocarbon aldehydes and carbonyl compounds, which contribute to the flavor and aroma of meat [62]. They also play a role in the formation of secondary lipid oxidation products such as malondialdehyde [63,64,65,66]. The antioxidant properties of phenolics in EOs are often attributed to the presence of hydroxyl groups, due to their ability to donate hydrogen atoms, stabilize free radicals, and prevent their degradation to more reactive oxidizing compounds such as malondialdehyde [32,43,44].

In this study, the effects of different concentrations of POEO on the TBARS values of beef are shown in Figure 5. In general, the duration of storage has a significant effect on the lipid oxidation of the meat, resulting in a significant increase in TBARS over 14 days at 4 °C. The TBARS levels of the POEO-treated samples were lower than those of the BHT-treated meat and the negative control, indicating the ability of these substances to inhibit lipid oxidation processes.

The most favorable results were obtained with 4POEO, which had a remarkably low value of 0.63 ± 0.03 mg MDA/kg meat on day 14. This result is well below the threshold value of 2 mg MDA/kg meat determined by Zhang et al. [67], indicating an excellent antioxidant effect and robust preservation of meat quality.

### 3.6. Changes in pH of Raw Ground Beef Samples

**Figure 6:**
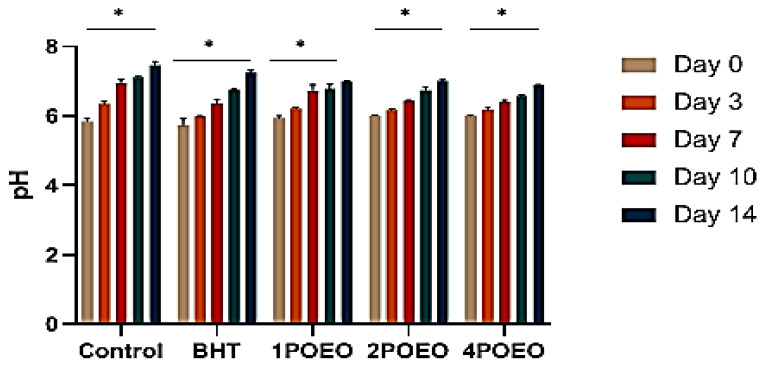
pH evolution of raw ground beef during refrigerated storage for 14 days. Values are the mean of three individual replicates (means ± SD). * *p* ≤ 0.05 for the same concentration are significantly different.

The pH of meat can be influenced by various factors, such as temperature, packaging, and the presence of microorganisms. The pH of the tested samples was not statistically influenced by the antioxidants contained in the POEO (Figure 6).

Similarly, in a study on the pH value of beef, the addition of lutein, olive leaf extract, ellagic acid, and sesame had no effect on pH [68].

Figure 6 shows the effects of different concentrations of POEO on the pH of meat samples after 14 days of storage. The pH values ranged from 5.8 to 6.02. The initial pH values of all treatments did not differ significantly, while they all increased progressively towards the end of storage. After 14 days, the effect of 4POEO was present but did not differ significantly from the control. During cold storage of meat, the pH value increases due to a combination of biochemical and microbial processes. The endogenous enzymes continue to break down proteins and release amino acids with basic side chains, which contribute to the increase in pH. In addition, microbial activity, even if slowed by refrigeration, can still generate alkaline by-products such as ammonia during growth and metabolism [69]. The formation of alkaline compounds from the degradation of amino acids and nucleic acids also contributes to the increase in pH. In addition, the loss of dissolved carbon dioxide, which contributes to the initial acidity of the meat, and changes in buffering capacity also play a role in increasing pH over time. Monitoring pH changes during storage is crucial for assessing meat quality and safety and for understanding the progression of biochemical and microbial reactions.

### 3.7. Effect of POEO on Sensory Attributes of Raw Ground Beef Samples

**Figure 7:**
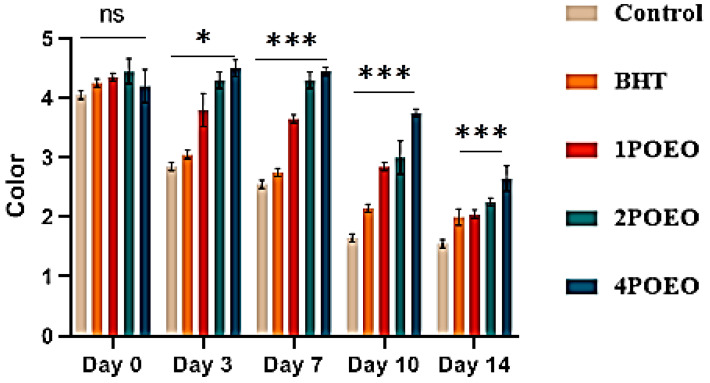
Evolution of sensory analyses during refrigerated storage at 4° C; for color. Values are the mean of three individual replicates (means ± SD). Groups (BHT, 1POEO, 2POEO, 4POEO) vs. group (control): *** *p* ≤ 0.001, * *p* ≤ 0.05. (ns) non-significant.

**Figure 8:**
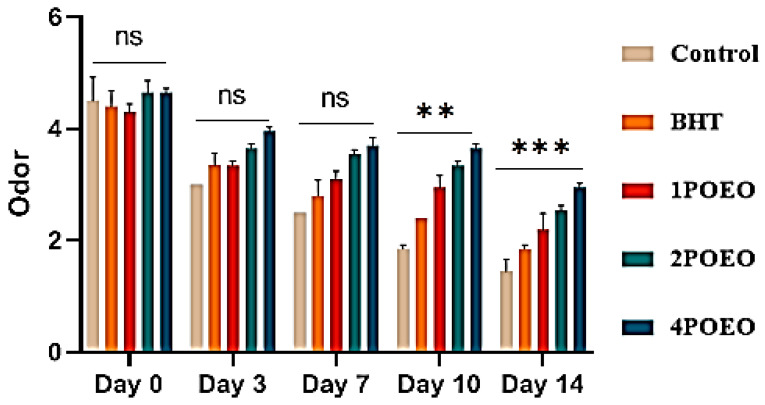
Evolution of sensory analyses during refrigerated storage at 4 °C; for odor. Values are the mean of three individual replicates (means ± SD). Groups (BHT, 1POEO, 2POEO, 4POEO) vs. group (control): *** *p* ≤ 0.001, ** *p* ≤ 0.01. (ns) non-significant.

**Figure 9:**
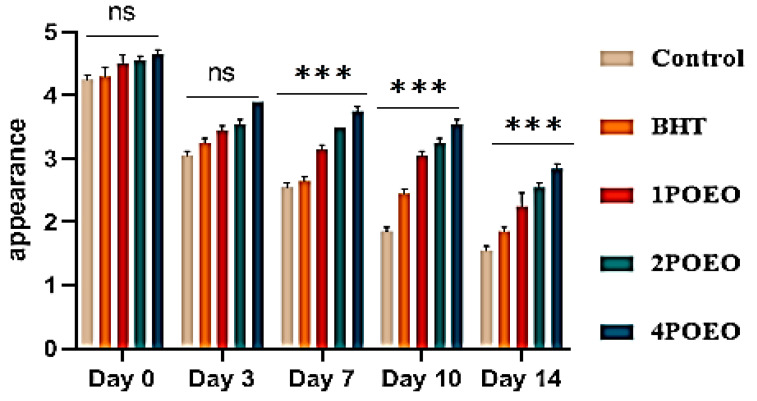
Evolution of sensory analyses during refrigerated storage at 4 °C; for appearance. Values are the mean of three individual replicates (means ± SD). Groups (BHT, 1POEO, 2POEO, 4POEO) vs. group (control): *** *p* ≤ 0.001. (ns) non-significant.

**Figure 10:**
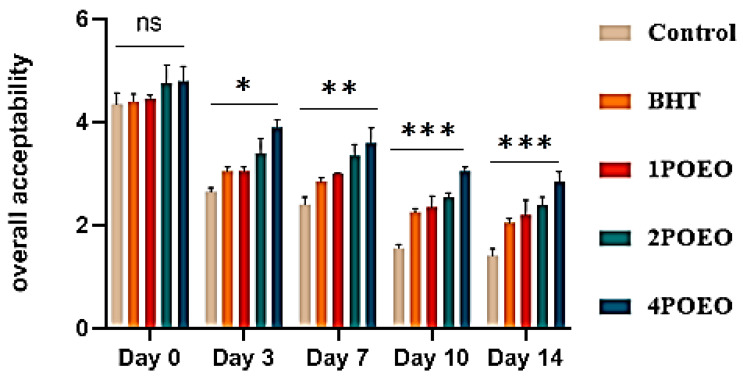
Evolution of sensory analyses during refrigerated storage at 4 °C; for overall acceptability. Values are the mean of three individual replicates (means ± SD). Groups (BHT, 1POEO, 2POEO, 4POEO) vs. group (control): *** *p* ≤ 0.001, ** *p* ≤ 0.01, * *p* ≤ 0.05. (ns) non-significant.

Ground beef samples to which different POEO concentrations were added were evaluated for different sensory properties. The results are shown in Figure 7, Figure 8, Figure 9 and Figure 10.

With increasing storage time, the sensory scores for all sensory attributes decreased significantly in all test groups (*p* < 0.05). At the end of storage, significant differences (*p* < 0.05) were found in the evaluation of all attributes between the control and POEO-treated samples. The mean average scores for color, appearance, odor, and overall acceptability of the 4POEO samples reached the highest values during the test storage period, followed by those of the 2POEO and then the 1POEO samples at the same level as BHT. As expected, the control product achieved the lowest scores for all attributes, ranging from 1.5 to 1.8 on the hedonic scale after 14 days of storage. The mean appearance values for control, BHT, 1POEO, 2POEO, and 4POEO from day 0 of cold storage were 3.6, 3.9, 4.8, 4.4, and 4.3, respectively. A significant decrease (*p* < 0.05) in the sensory properties of the control products was observed from the third day of storage, while the treated products showed a significant decrease (*p* < 0.05) from the tenth day.

The existing literature shows that samples treated with natural preservatives, especially essential oils (EO), exhibited better tenderness, color, aroma, and flavor compared to control samples [27]. The addition of essential oils also improved the overall acceptability of the samples, especially towards the end of the storage period, resulting in consumer preference [10,59,70,71]. Recent studies have highlighted the benefits of adding various EO such as rosemary, thyme, oregano, basil, coriander, ginger, garlic, cloves, juniper, and fennel, which when used individually or in combination, lead to improved sensory properties and a longer shelf life of meat and meat products [6,8,17,19,26,46].

### 3.8. Principal Component Analysis (PCA)

**Figure 11:**
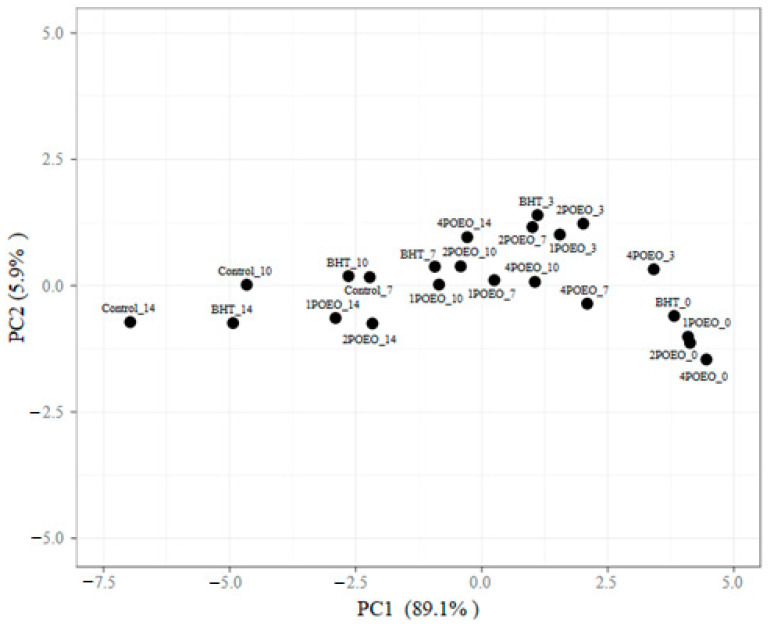
Principal component analysis (PCA) score plots derived from variables of raw ground beef treated with POEO during refrigerated storage for 14 days.

PCA was employed for multivariate statistical analysis of five samples with varying quality and storage duration during cold storage. PCA1 and PCA2 exhibited the highest eigenvalues with 89.1% and 5.91% of the variance, respectively, and together explained 94.6% of the total variability. Figure 11 illustrates the impact of quality and storage time on these samples.

The shift of the samples (dots) from right to left in the PCA score diagram indicates continuous changes in chemical, microbiological, and sensory properties during the storage period. The POEO treatment significantly inhibits the shift of the treated samples to the left, in contrast to the control. The PCA results show the effectiveness of POEO in maintaining the quality of ground beef, with 4POEO extending the shelf life of the meat by at least 10 days.

In the same case, the effect of *Salvia* EOs on meat quality was analyzed using PCA, which helped to investigate the relationships between different meat samples treated with EOs of *Salvia officinalis* and *Salvia sclarea* over different time periods and provide insight into how these oils affect meat quality characteristics over time [38]. EOs in marinades have also been linked to increased tenderness of poultry, pork, and beef, with PCA showing relationships between the type of essential oil used and meat quality after marinating [72]. In addition, research on protocatechuic acid has shown that it can reduce lipid oxidation, and PCA can be used to evaluate its effects on the oxidative stability of treated meat [73]. In addition, studies using rosemary and basil essential oils have shown an improvement in the microbial quality of raw ground meat, with PCA analyzing the effectiveness of these essential oils in meat preservation [74]. Finally, studies on consumer acceptance of beef from animals supplemented with essential oils indicated positive outcomes, with PCA employed to assess various quality variables, offering deeper insights into consumer preferences regarding meat quality influenced by essential oils [75].

### 3.9. Heatmap

This visual representation of a heat map shows the effects of different treatments on various quality and safety characteristics of a food product. Parameters evaluated include sensory aspects (odor, color, appearance, overall acceptability), chemical properties (pH, MetMb, TBARS), and microbial indicators (*Salmonella*, PTC, *Enterobacter*, aerobic plate count (APC)). The color scale from blue to red indicates the extent of the change, with blue representing lower and red higher values. It is noteworthy that treatments with “BHT” and “4POEO” seem to significantly reduce microbial growth (e.g., *Enterobacter* and APC), while some other treatments show high MetMb values, which could indicate oxidation or spoilage.

**Figure 12:**
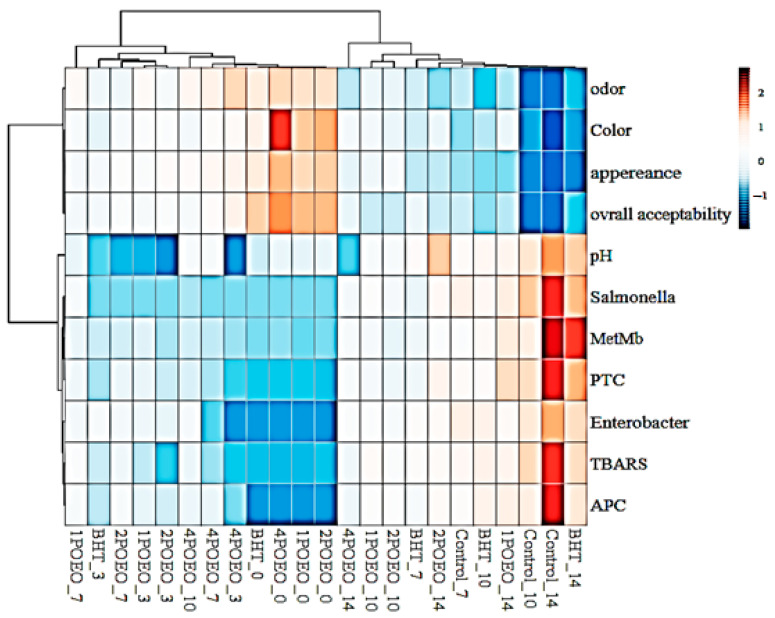
Heat map illustrating the correlation between meat quality parameters and samples over 14 days at 4 °C.

The dendrogram showed several correlations between the analyzed parameters. In fact, MetMb was negatively correlated with sensory analysis data, including color. Wang et al. [76,77,78] reported a clear causal relationship between meat color stability and myoglobin oxidation. Structural and chemical changes in myoglobin due to oxidative reactions are the main cause of meat color loss. In addition, Figure 12 shows that TBARS directly affects sensory characteristics, microbial growth, and pH.

Ben Akacha et al. [35] pointed out the significant impact of oxidative reactions on sensory properties, leading to lower consumer acceptance and the development of undesirable flavors. This phenomenon could be related to the high susceptibility of the phospholipid fraction to oxidation [27]. Langroodi et al. [71] found that sensory properties are closely related to microbiological evaluation. The presence of high levels of microorganisms and lipid oxidation are reflected in the negative sensory characteristics of the meat, such as unpleasant odor, discoloration, and poor visual appearance, as shown in Figure 8. In addition, an increased number of microorganisms, together with primary and secondary lipid oxidation and protein oxidation, were found to correlate with sensory characteristics. These factors are interrelated and contribute to the overall quality of the meat. In general, chemometric methods are valuable for linking color characteristics and oxidative stability to the authenticity and quality of meat during storage [53].

## 4. Conclusions

The findings of this study suggest that POEO has significant antioxidant and antibacterial activity thanks to its chemical composition and that it can be incorporated into minced meat to improve its physicochemical and microbiological properties. The study recommends the addition of at least 3.7, 7.4, or 14.8% of POEO to ground beef to effectively improve its shelf life (up to 20 days at 4 °C), stability, and quality. In summary, POEO has the potential to serve as a natural preservative, improve microbiological safety, and extend the shelf life of meat products, thus offering a promising alternative to synthetic preservatives.

## Figures and Tables

**Table 1:** Chemical profile (mean values ± SD) of *Pelargonium odoratissimum* essential oil (POEO).

N°	Component ^1^	LRI ^2^	LRI ^3^	POEO (%)
1	*β*-thujene	1120	1117	0.3 ± 0.02
2	*β*-myrcene	1170	1166	0.3 ± 0.02
3	*α*-phellandrene	1169	1177	0.1 ± 0.00
4	limonene	1200	1204	0.1 ± 0.00
5	*cis*-*β*-ocimene	1238	1243	0.2 ± 0.02
6	*trans-β*-ocimene	1265	1276	0.1 ± 0.00
7	*trans*-rose oxide	1378	1383	0.8 ± 0.02
8	isomenthone	1480	1484	7.4 ± 0.07
9	linalool	1531	1529	4.8 ± 0.05
10	isopinocamphone	1533	1530	0.1 ± 0.02
11	(-)-*β*-bourbonene	1538	1540	0.6 ± 0.02
12	(-)-aristolene	1570	1565	9.8 ± 0.05
13	citronellyl formate	1600	1605	12.6 ± 0.08
14	citronellol acetate	1637	1644	1.0 ± 0.02
15	humulene	1671	1667	0.3 ± 0.02
16	citronellol	1728	1730	40.0 ± 0.14
17	*α*-selinene	1748	1744	0.3 ± 0.02
18	citronellyl butyrate	1791	1786	1.3 ± 0.02
19	nerol	1793	1798	15.3 ± 0.07
20	geranyl isobutyrate	1811	1819	2.6 ± 0.03
21	geranyl proprionate	1827	1830	0.4 ± 0.02
22	citronellyl tiglate	1989	1991	0.7 ± 0.03
23	cubenol	2027	2030	0.5 ± 0.0.2
24	phenyl ethyl tiglate	2185	2190	0.3 ± 0.02
25	geranic acid	2360	2357	0.1 ± 0.01
	SUM			100.0
	Monoterpenes			6.8
	Oxygenated monoterpenes			81.2
	Sesquiterpenes			11.0
	Others			1.0

^1^ the components are reported according to their elution order on a polar column; ^2^ Linear Retention Indices calculated using the polar column; ^3^ Linear Retention indices from literature.

**Table 2:** Antibacterial activity of *Pelargonium odoratissimum* essential oil (POEO) against food-contaminating bacteria expressed as inhibition diameters (mm).

Tested EO	*Pelargonium odoratissimum*		Carbeniciline	
	Diameter of inhibition (mm)			
Concentrations	25 (mg/mL)	50 (mg/mL)	10 (µg/mL)	5 (µg/mL)
Gram-positive				
*B. cereus* ATCC 14579	-	-	24 ± 0.1	18 ± 0.03
*S. aureus* ATCC 25923	13.0 ± 0.00	15.0 ± 0.00	22 ± 0.00	14 ± 0.02
*E. faecalis* ATCC 29212	14.5 ± 0.21	27.5 ± 0.49	29 ± 0.34	16 ± 0.44
*M. luteus* ATCC 1880	19.0 ± 0.00	29.0 ± 0.00	22 ± 0.33	19 ± 0.24
*L. monocytogenes* ATCC 1911	-	21.0 ± 0.00	22 ± 0.17	13 ± 0.84
Gram-negative				
*P. aeruginosa* ATCC 9027	-	-	23 ± 0.07	14 ± 0.01
*E. coli* ATCC 25922	16.0 ± 0.00	26.0 ± 0.00	31 ± 0.05	18 ± 0.04
*S. enterica* ATCC 43972	-	-	22 ± 0.05	12 ± 0.02

Values are given as mean ± S.D. of a triplicate experiment.

**Table 3:** Evaluation of minimum inhibitory concentration (MIC, mg/mL) and minimum bactericidal concentration (MBC, mg/mL) of *Pelargonium odoratissimim* essential oil against pathogenic bacteria.

Bacterial Strains	MIC	MBC	MBC/MIC	Interpretation
Gram-positive				
*B. cereus* ATCC 14579 *S. aureus* ATCC 25923	9.35 ± 7.99 9.35 ± 7.99	9.35 ± 7.99 11.25 ± 5.30	1 1	Bactericidal Bactericidal
*E. faecalis* ATCC 29212 *M. luteus* ATCC 1880	5.6 ± 2.68 8.25 ± 9.54	9.35 ± 7.99 8.25 ± 9.54	2 1	Bacteriostatic Bactericidal
*L. monocytogenes* ATCC 1911	9.35 ± 7.99	11.25 ± 5.30	1	Bactericidal
Gram-negative				
*P. aeruginosa* ATCC 9027	-	-	-	
*E. coli* ATCC 25922	8.25 ± 9.54	11.25 ± 5.30	1	Bactericidal
*S. enterica* ATCC 43972	9.35 ± 7.99	11.25 ± 5.30	1	Bactericidal
	Carbenicillin			
Gram-positive				
*B. cereus* ATCC 14579 *S. aureus* ATCC 25923	3.125 ± 2.65 1.875 ± 0.88	0.0 0.0	2 2	Bactericidal Bactericidal
*E. faecalis* ATCC 29212 *M. luteus* ATCC 1880	0.925 ± 0.45 2.500 ± 0.00	0.0 0.0	2 2	Bactericidal Bactericidal
*L. monocytogenes* ATCC 1911	3.125 ± 2.65	0.0	4	Bacteriostatic
Gram-negative				
*P. aeruginosa* ATCC 9027	1.875 ± 0.88	0.0	1	Bactericidal
*E. coli* ATCC 25922	0.925 ± 0.45	0.0	6	Bacteriostatic
*S. enterica* ATCC 43972	1.250 ± 0.00	0.0	3	Bacteriostatic

Values are means (*n* = 3).

**Table 4:** Effect of POEO on Enterobacteriaceae and *Salmonella* spp. (log_10_ CFU/g) in raw minced beef meat during 14 days of cold storage.

	Refrigerated Storage Days at 4 °C. (log_10_ CFU/g)				
**Enterobacteriaceae**	0	3	7	10	14
Control	<1 ± 0.00 ^aA^	2.29 ± 0.05 ^bC^	2.57 ± 0.03 ^cC^	2.72 ± 0.03 ^dD^	3.18 ± 0.20 ^eD^
BHT	<1 ± 0.00 ^aA^	2.08 ± 0.02 ^bB^	2.16 ± 0.07 ^bcBC^	2.53 ± 0.01 ^cC^	2.84 ± 0.05 ^cC^
1POEO	<1 ± 0.00 ^aA^	1.87 ± 0.007 ^bB^	1.90 ± 0.05 ^bB^	2.17 ± 0.07 ^cB^	2.32 ± 0.02 ^cB^
2POEO	<1 ± 0.00 ^aA^	1.69 ± 0.003 ^aB^	1.95 ± 0.04 ^bB^	2.04 ± 0.03 ^bB^	2.25 ± 0.007 ^bB^
4POEO	<1 ± 0.00 ^aA^	1.08 ± 0.08 ^aA^	1.36 ± 0.04 ^aA^	1.98 ± 0.01 ^aA^	2.10 ± 0.04 ^bA^
***Salmonella* spp**.	0	3	7	10	14
Control	<1 ± 0.00 ^aA^	<1 ± 0.00 ^aA^	1.68 ± 0.02 ^bC^	1.84 ± 0.06 ^cD^	2.20 ± 0.04 ^eD^
BHT	<1 ± 0.00 ^aA^	<1 ± 0.00 ^aA^	1.21 ± 0.001 ^bB^	1.55 ± 0.008 ^cC^	2.02 ± 0.16 ^cC^
1POEO	<1 ± 0.00 ^aA^	<1 ± 0.00 ^aA^	1.33 ± 0.04 ^bB^	1.42 ± 0.03 ^cB^	1.74 ± 0.03 ^cB^
2POEO	<1 ± 0.00 ^aA^	<1 ± 0.00 ^aA^	<1 ± 0.00 ^bB^	1.34 ± 0.01 ^bB^	1.49 ± 0.06 ^bB^
4POEO	<1 ± 0.00 ^aA^	<1 ± 0.00 ^aA^	<1 ± 0.00 ^aA^	1.03 ± 0.01 ^aA^	1.32 ± 0.01 ^bA^

Means ± SEM (*n = 3*). Values with a different letter (a–e) for the same storage day are significantly different (*p* < 0.05). Values with a different letter (A–D) for the same concentration are significantly different.

## Data Availability

The original contributions presented in the study are included in the article, further inquiries can be directed to the corresponding author.

## References

[1] ur Rahman U., Sahar A., Ishaq A., Aadil R.M., Zahoor T., Ahmad M.H. (2018). Advanced Meat Preservation Methods: A Mini Review. J. Food Saf..

[2] Sridhar A., Ponnuchamy M., Kumar P.S., Kapoor A. (2021). Food Preservation Techniques and Nanotechnology for Increased Shelf Life of Fruits, Vegetables, Beverages and Spices: A Review. Environ. Chem. Lett..

[3] Rudy M., Kucharyk S., Duma-Kocan P., Stanisławczyk R., Gil M. (2020). Unconventional Methods of Preserving Meat Products and Their Impact on Health and the Environment. Sustainability.

[4] Kaale L.D., Eikevik T.M., Rustad T., Kolsaker K. (2011). Superchilling of Food: A Review. J. Food Eng..

[5] Taieb Bouteraa M., Ben Akacha B., Kačániová M., Čmiková N., Ben Romdhane W., Baazaoui N., Soltani N., Chouaibi Y., Ben Hsouna A., Garzoli S. (2024). Exploring the Antimicrobial and Antioxidant Properties of TdGASA2 Protein: From Molecular Insights to a Promising Natural Preservative for Tunisian Cheese Shelf-Life Enhancement. Food Biosci..

[6] Jha P.K., Sadot M., Vino S.A., Jury V., Curet-Ploquin S., Rouaud O., Havet M., Le-Bail A. (2017). A Review on Effect of DC Voltage on Crystallization Process in Food Systems. Innov. Food Sci. Emerg. Technol..

[7] Soyer A., Özalp B., Dalmış Ü., Bilgin V. (2010). Effects of Freezing Temperature and Duration of Frozen Storage on Lipid and Protein Oxidation in Chicken Meat. Food Chem..

[8] Ben Akacha B., Michalak M., Ben Romdhane W., Kačániová M., Ben Saad R., Mnif W., Kukula-Koch W., Garzoli S., Ben Hsouna A. (2024). Recent Advances in Phytochemistry, Pharmaceutical, Biomedical, Phytoremediation, and Bio-Preservative Applications of *Lobularia maritima*. S. Afr. J. Bot..

[9] Boler D.D., Woerner D.R. (2017). What Is Meat? A Perspective from the American Meat Science Association. Anim. Front..

[10] Fratianni F., De Martino L., Melone A., De Feo V., Coppola R., Nazzaro F. (2010). Preservation of Chicken Breast Meat Treated with Thyme and Balm Essential Oils. J. Food Sci..

[11] Gómez I., Janardhanan R., Ibañez F.C., Beriain M.J. (2020). The Effects of Processing and Preservation Technologies on Meat Quality: Sensory and Nutritional Aspects. Foods.

[12] Sun S., Rasmussen F.D., Cavender G.A., Sullivan G.A. (2019). Texture, Color and Sensory Evaluation of Sous-Vide Cooked Beef Steaks Processed Using High Pressure Processing as Method of Microbial Control. LWT.

[13] Chaari M., Elhadef K., Akermi S., Ben Akacha B., Fourati M., Chakchouk Mtibaa A., Ennouri M., Sarkar T., Shariati M.A., Rebezov M. (2022). Novel Active Food Packaging Films Based on Gelatin-Sodium Alginate Containing Beetroot Peel Extract. Antioxidants.

[14] Ben Akacha B., Garzoli S., Ben Saad R., Brini F., Mnif W., Kačániová M., Ben Hsouna A. (2023). Biopreservative Effect of the Tunisian Halophyte *Lobularia maritima* Flavonoid Fraction, Used Alone and in Combination with Linalool in Stored Minced Beef Meat. Metabolites.

[15] Ben Akacha B., Michalak M., Najar B., Venturi F., Taglieri I., Kačániová M., Ben Saad R., Mnif W., Garzoli S., Ben Hsouna A. (2023). Recent Advances in the Incorporation of Polysaccharides with Antioxidant and Antibacterial Functions to Preserve the Quality and Shelf Life of Meat Products. Foods.

[16] Goudarzi J., Moshtaghi H., Shahbazi Y. (2023). Kappa-Carrageenan-Poly(Vinyl Alcohol) Electrospun Fiber Mats Encapsulated with *Prunus domestica* Anthocyanins and Epigallocatechin Gallate to Monitor the Freshness and Enhance the Shelf-Life Quality of Minced Beef Meat. Food Packag. Shelf Life.

[17] Fernández-López J., Viuda-Martos M. (2018). Introduction to the Special Issue: Application of Essential Oils in Food Systems. Foods.

[18] Prakash B., Kedia A., Singh A., Yadav S., Singh A., Yadav A., Deepika, Dubey N.K. (2016). Antifungal, Antiaflatoxin and Antioxidant Activity of Plant Essential Oils and Their In Vivo Efficacy in Protection of Chickpea Seeds: Essential Oils as Food Preservatives. J. Food Qual..

[19] Bhavaniramya S., Vishnupriya S., Al-Aboody M.S., Vijayakumar R., Baskaran D. (2019). Role of Essential Oils in Food Safety: Antimicrobial and Antioxidant Applications. Grain Oil Sci. Technol..

[20] Hsouna A.B., Čmiková N., Akacha B.B., Saad R.B., Mnif W., Garzoli S., Kačániová M. (2024). Changes in Inoculated *Salmonella enterica* subsp. *enterica* Serovar Enteritidis and Other Microbiological Qualities of Vacuum-Packed Carrot Slices after Treatment with Aqueous Extract of *Lobularia maritima*. Heliyon.

[21] Ju J., Chen X., Xie Y., Yu H., Guo Y., Cheng Y., Qian H., Yao W. (2019). Application of Essential Oil as a Sustained Release Preparation in Food Packaging. Trends Food Sci. Technol..

[22] Pandey A.K., Kumar P., Singh P., Tripathi N.N., Bajpai V.K. (2017). Essential Oils: Sources of Antimicrobials and Food Preservatives. Front. Microbiol..

[23] Yu H.H., Chin Y.-W., Paik H.-D. (2021). Application of Natural Preservatives for Meat and Meat Products against Food-Borne Pathogens and Spoilage Bacteria: A Review. Foods.

[24] Aguiar Campolina G., das Graças Cardoso M., Rodrigues-Silva-Caetano A., Lee Nelson D., Mendes Ramos E. (2023). Essential Oil and Plant Extracts as Preservatives and Natural Antioxidants Applied to Meat and Meat Products: A Review. Food Technol. Biotechnol..

[25] Froiio F., Mosaddik A., Morshed M.T., Paolino D., Fessi H., Elaissari A. (2019). Edible Polymers for Essential Oils Encapsulation: Application in Food Preservation. Ind. Eng. Chem. Res..

[26] Lisboa H.M., Pasquali M.B., dos Anjos A.I., Sarinho A.M., de Melo E.D., Andrade R., Batista L., Lima J., Diniz Y., Barros A. (2024). Innovative and Sustainable Food Preservation Techniques: Enhancing Food Quality, Safety, and Environmental Sustainability. Sustainability.

[27] Ben Akacha B., Švarc-Gajić J., Elhadef K., Ben Saad R., Brini F., Mnif W., Smaoui S., Ben Hsouna A. (2022). The Essential Oil of Tunisian Halophyte *Lobularia maritima*: A Natural Food Preservative Agent of Ground Beef Meat. Life.

[28] Kačániová M., Vukic M., Vukovic N.L., Čmiková N., Verešová A., Schwarzová M., Babošová M., Porhajašová J.I., Kluz M., Waszkiewicz-Robak B. (2023). An In-Depth Study on the Chemical Composition and Biological Effects of *Pelargonium graveolens* Essential Oil. Foods.

[29] Andrade M.A., Cardoso M.G., Batista L.R., Freire J.M., Nelson D.L. (2011). Antimicrobial Activity and Chemical Composition of Essential Oil of *Pelargonium odoratissimum*. Rev. Bras. Farmacogn..

[30] Ben Akacha B., Michalak M., Generalić Mekinić I., Kačániová M., Chaari M., Brini F., Ben Saad R., Mnif W., Garzoli S., Ben Hsouna A. (2024). Mixture Design of α-Pinene, α-Terpineol, and 1,8-Cineole: A Multiobjective Response Followed by Chemometric Approaches to Optimize the Antibacterial Effect against Various Bacteria and Antioxidant Activity. Food Sci. Nutr..

[31] Al-Mijalli S.H., Assaggaf H., Qasem A., El-Shemi A.G., Abdallah E.M., Mrabti H.N., Bouyahya A. (2022). Antioxidant, Antidiabetic, and Antibacterial Potentials and Chemical Composition of *Salvia officinalis* and *Mentha suaveolens* Grown Wild in Morocco. Adv. Pharmacol. Pharm. Sci..

[32] Sebei K., Sakouhi F., Herchi W., Khouja M.L., Boukhchina S. (2015). Chemical Composition and Antibacterial Activities of Seven *Eucalyptus* Species Essential Oils Leaves. Biol. Res..

[33] Parvekar P., Palaskar J., Metgud S., Maria R., Dutta S. (2020). The Minimum Inhibitory Concentration (MIC) and Minimum Bactericidal Concentration (MBC) of Silver Nanoparticles against *Staphylococcus aureus*. Biomater. Investig. Dent..

[34] Zakaria Nabti L., Sahli F., Laouar H., Olowo-okere A., Nkuimi Wandjou J.G., Maggi F. (2020). Chemical Composition and Antibacterial Activity of Essential Oils from the Algerian Endemic *Origanum glandulosum* Desf. against Multidrug-Resistant Uropathogenic *E. coli* Isolates. Antibiotics.

[35] Akacha B.B., Najar B., Venturi F., Quartacci M.F., Saad R.B., Brini F., Mnif W., Kačániová M., Ben Hsouna A. (2022). A New Approach in Meat Bio-Preservation through the Incorporation of a Heteropolysaccharide Isolated from *Lobularia maritima* L. Foods.

[36] Peng Z., Li Y., Yan L., Yang S., Yang D. (2023). Correlation Analysis of Microbial Contamination and Alkaline Phosphatase Activity in Raw Milk and Dairy Products. Int. J. Environ. Res. Public Health.

[37] Xiao T., Li Y., Hu L., Nie P., Ramaswamy H.S., Yu Y. (2022). Demonstration of *Escherichia coli* Inactivation in Sterile Physiological Saline under High Pressure (HP) Phase Transition Conditions and Analysis of Probable Contribution of HP Metastable Positions Using Model Solutions and Apple Juice. Foods.

[38] Ben Akacha B., Ben Hsouna A., Generalić Mekinić I., Ben Belgacem A., Ben Saad R., Mnif W., Kačániová M., Garzoli S. (2023). *Salvia officinalis* L. and Salvia Sclarea Essential Oils: Chemical Composition, Biological Activities and Preservative Effects against *Listeria monocytogenes* Inoculated into Minced Beef Meat. Plants.

[39] Krzywicki K. (1982). The Determination of Haem Pigments in Meat. Meat Sci..

[40] Elhadef K., Smaoui S., Ben Hlima H., Ennouri K., Fourati M., Chakchouk Mtibaa A., Ennouri M., Mellouli L. (2020). Effects of *Ephedra alata* Extract on the Quality of Minced Beef Meat during Refrigerated Storage: A Chemometric Approach. Meat Sci..

[41] Eymard S., Carcouët E., Rochet M., Dumay J., Chopin C., Genot C. (2005). Development of Lipid Oxidation during Manufacturing of Horse Mackerel Surimi. J. Sci. Food Agric..

[42] Ara K., Hama M., Akiba S., Koike K., Okisaka K., Hagura T., Kamiya T., Tomita F. (2006). Foot Odor Due to Microbial Metabolism and Its Control. Can. J. Microbiol..

[43] Wu Y., OuYang Q., Tao N. (2016). Plasma Membrane Damage Contributes to Antifungal Activity of Citronellal against *Penicillium digitatum*. J. Food Sci. Technol..

[44] Kaura T., Pervaiz N., Mewara A., Martin C.R., Hollins Martin C.J., Preedy V.R., Rajendram R. (2021). Chapter 32—Larvicides: Plant Oils and Zika Control. Zika Virus Impact, Diagnosis, Control, and Models.

[45] Zhou A.-A., Li R.-Y., Mo F.-X., Ding Y., Li R.-T., Guo X., Hu K., Li M. (2022). Natural Product Citronellal Can Significantly Disturb Chitin Synthesis and Cell Wall Integrity in *Magnaporthe oryzae*. J. Fungi.

[46] Ramezani H., Singh H.P., Batish D.R., Kohli R.K., Dargan J.S. (2002). Fungicidal Effect of Volatile Oils from *Eucalyptus citriodora* and Its Major Constituent Citronellal. N. Z. Plant Prot..

[47] Lu J.-X., Guo C., Ou W.-S., Jing Y., Niu H.-F., Song P., Li Q.-Z., Liu Z., Xu J., Li P. (2019). Citronellal Prevents Endothelial Dysfunction and Atherosclerosis in Rats. J. Cell. Biochem..

[48] Fernandez-Soto P., Celi D., Tejera E., Alvarez-Suarez J.M., Machado A. (2023). *Cinnamomum* sp. and *Pelargonium odoratissimum* as the Main Contributors to the Antibacterial Activity of the Medicinal Drink Horchata: A Study Based on the Antibacterial and Chemical Analysis of 21 Plants. Molecules.

[49] Nazzaro F., Fratianni F., De Martino L., Coppola R., De Feo V. (2013). Effect of Essential Oils on Pathogenic Bacteria. Pharmaceuticals.

[50] Bouyahya A., Bakri Y., Et-Touys A., Talbaoui A., Khouchlaa A., Charfi S., Abrini J., Dakka N. (2017). Résistance aux antibiotiques et mécanismes d’action des huiles essentielles contre les bactéries. Phytothérapie.

[51] Abdelbaky A.S., Abd El-Mageed T.A., Babalghith A.O., Selim S., Mohamed A.M.H.A. (2022). Green Synthesis and Characterization of ZnO Nanoparticles Using *Pelargonium odoratissimum* (L.) Aqueous Leaf Extract and Their Antioxidant, Antibacterial and Anti-Inflammatory Activities. Antioxidants.

[52] Jayaraj R.L., Azimullah S., Parekh K.A., Ojha S.K., Beiram R. (2022). Effect of Citronellol on Oxidative Stress, Neuroinflammation and Autophagy Pathways in an in Vivo Model of Parkinson’s Disease. Heliyon.

[53] Chaudhary P., Janmeda P., Docea A.O., Yeskaliyeva B., Abdull Razis A.F., Modu B., Calina D., Sharifi-Rad J. (2023). Oxidative Stress, Free Radicals and Antioxidants: Potential Crosstalk in the Pathophysiology of Human Diseases. Front. Chem..

[54] Papadochristopoulos A., Kerry J.P., Fegan N., Burgess C.M., Duffy G. (2021). Natural Anti-Microbials for Enhanced Microbial Safety and Shelf-Life of Processed Packaged Meat. Foods.

[55] Veselá H., Kameník J., Dušková M., Ježek F., Svobodová H. (2024). Effect of Dry Aging of Pork on Microbiological Quality and Instrumental Characteristics. Foods.

[56] Malavi D.N., Muzhingi T., Abong’ G.O. (2018). Good Manufacturing Practices and Microbial Contamination Sources in Orange Fleshed Sweet Potato Puree Processing Plant in Kenya. Int. J. Food Sci..

[57] Quiñones J., Díaz R., Velázquez L., Martínez A., Sepúlveda G., Huaiquipán R., Short S., Velásquez C., Cancino D., Tapía D. (2024). *Durvillaea antarctica* Meal as a Possible Functional Ingredient in Traditional Beef Burgers. Appl. Sci..

[58] Jayasena D.D., Jo C. (2013). Essential Oils as Potential Antimicrobial Agents in Meat and Meat Products: A Review. Trends Food Sci. Technol..

[59] Shahbazi Y., Karami N., Shavisi N. (2018). Effect of *Mentha Spicata* Essential Oil on Chemical, Microbial, and Sensory Properties of Minced Camel Meat during Refrigerated Storage. J. Food Saf..

[60] Hsouna A.B., Boye A., Ackacha B.B., Dhifi W., Saad R.B., Brini F., Mnif W., Kačániová M. (2022). Thiamine Demonstrates Bio-Preservative and Anti-Microbial Effects in Minced Beef Meat Storage and Lipopolysaccharide (LPS)-Stimulated RAW 264.7 Macrophages. Animals.

[61] Bekhit A.E.D., Faustman C. (2005). Metmyoglobin Reducing Activity. Meat Sci..

[62] Bou R., Guardiola F., Codony R., Faustman C., Elias R.J., Decker E.A. (2008). Effect of Heating Oxymyoglobin and Metmyoglobin on the Oxidation of Muscle Microsomes. J. Agric. Food Chem..

[63] Van Haute S., Raes K., Van der Meeren P., Sampers I. (2016). The Effect of Cinnamon, Oregano and Thyme Essential Oils in Marinade on the Microbial Shelf Life of Fish and Meat Products. Food Control.

[64] Baron C.P., Andersen H.J. (2002). Myoglobin-Induced Lipid Oxidation. A Review. J. Agric. Food Chem..

[65] Pandey H., Kumar S. (2021). Butylated Hydroxytoluene and Butylated Hydroxyanisole Induced Cyto-Genotoxicity in Root Cells of *Allium cepa* L. Heliyon.

[66] Hyldgaard M., Mygind T., Meyer R. (2012). Essential Oils in Food Preservation: Mode of Action, Synergies, and Interactions with Food Matrix Components. Front. Microbiol..

[67] Zhang Y., Holman B.W.B., Ponnampalam E.N., Kerr M.G., Bailes K.L., Kilgannon A.K., Collins D., Hopkins D.L. (2019). Understanding Beef Flavour and Overall Liking Traits Using Two Different Methods for Determination of Thiobarbituric Acid Reactive Substance (TBARS). Meat Sci..

[68] Reitznerová A., Šuleková M., Nagy J., Marcinčák S., Semjon B., Čertík M., Klempová T. (2017). Lipid Peroxidation Process in Meat and Meat Products: A Comparison Study of Malondialdehyde Determination between Modified 2-Thiobarbituric Acid Spectrophotometric Method and Reverse-Phase High-Performance Liquid Chromatography. Mol. J. Synth. Chem. Nat. Prod. Chem..

[69] Atasoy M., Álvarez Ordóñez A., Cenian A., Djukić-Vuković A., Lund P.A., Ozogul F., Trček J., Ziv C., De Biase D. (2024). Exploitation of Microbial Activities at Low pH to Enhance Planetary Health. FEMS Microbiol. Rev..

[70] Li C., Zhang C., Chen X., Cui H., Lin L. (2022). The Interference Mechanism of Basil Essential Oil on the Cell Membrane Barrier and Respiratory Metabolism of Listeria Monocytogenes. Front. Microbiol..

[71] Mojaddar Langroodi A., Nematollahi A., Sayadi M. (2021). Chitosan Coating Incorporated with Grape Seed Extract and Origanum Vulgare Essential Oil: An Active Packaging for Turkey Meat Preservation. J. Food Meas. Charact..

[72] Siroli L., Baldi G., Soglia F., Bukvicki D., Patrignani F., Petracci M., Lanciotti R. (2020). Use of Essential Oils to Increase the Safety and the Quality of Marinated Pork Loin. Foods.

[73] Deuchande T., Fundo J.F., Pintado M.E., Amaro A.L. (2024). Protocatechuic Acid as an Inhibitor of Lipid Oxidation in Meat. Meat Sci..

[74] Befa Kinki A., Atlaw T., Haile T., Meiso B., Belay D., Hagos L., Hailemichael F., Abid J., Elawady A., Firdous N. (2024). Preservation of Minced Raw Meat Using Rosemary (*Rosmarinus officinalis*) and Basil (*Ocimum basilicum*) Essential Oils. Cogent Food Agric..

[75] Liu J., Ellies-Oury M.-P., Stoyanchev T., Hocquette J.-F. (2022). Consumer Perception of Beef Quality and How to Control, Improve and Predict It? Focus on Eating Quality. Foods.

[76] Wang Y., Zhang Y., Song X., Fang C., Xing R., Liu L., Zhao X., Zou Y., Li L., Jia R. (2022). 1,8-Cineole Inhibits Biofilm Formation and Bacterial Pathogenicity by Suppressing luxS Gene Expression in *Escherichia coli*. Front. Pharmacol..

[77] Maritha V., Harlina P.W., Musfiroh I., Gazzali A.M., Muchtaridi M. (2022). The Application of Chemometrics in Metabolomic and Lipidomic Analysis Data Presentation for Halal Authentication of Meat Products. Molecules.

[78] Boyaci İ.H., Uysal R.S., Temiz T., Shendi E.G., Yadegari R.J., Rishkan M.M., Velioglu H.M., Tamer U., Ozay D.S., Vural H. (2014). A Rapid Method for Determination of the Origin of Meat and Meat Products Based on the Extracted Fat Spectra by Using of Raman Spectroscopy and Chemometric Method. Eur. Food Res. Technol..

